# Supplementary material for: Computational phenotyping of brain-behavior dynamics underlying approach-avoidance conflict in major depressive disorder
Source: PLoS Comput Biol. 2021 May 10;17(5):e1008955. doi: 10.1371/journal.pcbi.1008955 (PMC8136861; doi:10.1371/journal.pcbi.1008955)
Supplement: S1 Text — Fig A. Selected results from the computational mixed-effect model. For each coefficient the left plot shows the group posterior distribution for healthy controls (HC) and individuals with major depressive disorder (MDD). The right plot shows the posterior distribution of difference as a measure of the effect of group on each coefficient, and the probability given data that the coefficient is higher in HC than MDD. A, weight of aversiveness onto drift rate (v), B, weight of reward onto drift rate (v), C, estimated relative starting point (z) between decision thresholds, D, impact of Pavlovian effect onto starting point (z), E, impact of activity in nucleus accumbens (NAcc) onto starting point (z), F, impact of activity in the pACC onto drift rate (v). The results from the mixed-effect model here overlap with the results from the model in Fig 3, in which the two groups were estimated separately. Table A. Description and fit of tested models. Model comparison was performed by comparing a baseline model to a model in which one ‘component’ was modified. The model we report from includes all the ‘components’ that improved fit compared to the baseline model. The function of the impact of reward and aversiveness onto drift rate was assumed to be linear or logarithmic, and were assessed on whether model fit was improved when including a dummy coded variable that indicated whether the offered value of reward (Dreward) or aversiveness (Daverse) was 0 (D = 1) or not (D = 0). Conflict was measured as the absolute difference in reward and aversiveness and was estimated to influence the decision threshold parameter. PavlovianBias included information on whether approaching (avoiding) offers involved pushing (pulling) the joystick to respond (PavlovianBias = 1) or vice-versa (PavlovianBias = 0). Lower values of DIC indicate better fit to data. DIC = deviance information criterion. Fig B. Observed (black) and predicted (red) response time distributions across subjects. Avoid-decisio [file pcbi.1008955.s001.docx]

S1 Text

Computational phenotyping of brain-behavior dynamics underlying approach-avoidance in major depressive disorder

## M. L. Pedersen^1,2,3.*^, M. Ironside^4,5^, K.-I. Amemori^6,7,8,9^, C.L. McGrath^4,5^, M.S. Kang^5^, A. M. Graybiel^6,7^, D. A. Pizzagalli^4,5,10,#^, M. J. Frank^1,2,#,*^

# ^1^ Department of Cognitive, Linguistic & Psychological Sciences, Brown University, Providence, RI, USA

# ^2^ Carney Institute for Brain Science, Brown University, Providence, RI, USA

# ^3^ Department of Psychology, University of Oslo, Oslo, Norway

# ^4^ Department of Psychiatry, Harvard Medical School, Boston, MA, USA

# ^5^ Center for Depression, Anxiety and Stress Research, McLean Hospital, Boston, MA, USA

^6^ Department of Brain and Cognitive Sciences, Massachusetts Institute of Technology, MA, USA

^7^ McGovern Institute for Brain Research, Massachusetts Institute of Technology, Cambridge, MA, USA

^8^ Hakubi Center for Advanced Research, Kyoto University, Kyoto, Japan

^9^ Primate Research Institute, Kyoto University, Aichi, Japan

# ^10^ McLean Imaging Center, McLean Hospital, Boston, MA, USA

# co-senior authors

* [madslupe@gmail.com](mailto:madslupe@gmail.com) (MLP), [Michael_frank@brown.edu](mailto:Michael_frank@brown.edu) (MJF)

## Supplementary Methods

## Sample information

Participants were right-handed, reported no medical or neurological illnesses and no current use of psychotropic medications. Healthy controls reported no current or past psychopathology. All participants were assessed by a clinician using the Structured Clinical Interview for the DSM-IV (SCID; (1)) and the Hamilton Depression Rating Scale (HAM-D, (2)). Participants were compensated $15/hour for their time, $50 for the MRI session and a $50 completion bonus.

During the initial screening visit, after the SCID, participants completed a number of self-report questionnaires, including the Beck Depression Inventory (BDI-II; (3)), Snaith Hamilton Pleasure Scale (SHPS; (4)), the Cognitive-Behavioral Avoidance Scale (CBAS; (5)), the Mood and Anxiety Symptom Questionnaire (MASQ; (6)) and the Perceived Stress Scale (PSS; (7)) in order to assess, respectively, depressive symptoms, anhedonic symptoms, behavioral avoidance, subtypes of depressive and anxious symptoms, and perceived stress.

**Fig A.** Selected results from the computational mixed-effect model. For each coefficient the left plot shows the group posterior distribution for healthy controls (HC) and individuals with major depressive disorder (MDD). The right plot shows the posterior distribution of difference as a measure of the effect of group on each coefficient, and the probability given data that the coefficient is higher in HC than MDD. **A**, weight of aversiveness onto drift rate (v), **B**, weight of reward onto drift rate (v), **C**, estimated relative starting point (z) between decision thresholds, **D**, impact of Pavlovian effect onto starting point (z), **E**, impact of activity in nucleus accumbens (NAcc) onto starting point (z), **F**, impact of activity in the pACC onto drift rate (v). The results from the mixed-effect model here overlap with the results from the model in Figure 3, in which the two groups were estimated separately.

**Table A.** Description and fit of tested models.

| **Model** | **Reward** | **Averse** | **Dreward** | **Daverse** | **Conflict** | **PavlovianBias** | **DIC** |
| --- | --- | --- | --- | --- | --- | --- | --- |
| baseline | linear | linear | no | no | no | no | 7544 |
| Log(reward) | log | linear | no | no | no | no | 7214 |
| Log(averse) | linear | log | no | no | no | no | 7569 |
| Dreward | linear | linear | yes | no | no | no | 7023 |
| Daverse | linear | linear | no | yes | no | no | 7553 |
| Conflict | linear | linear | no | no | yes | no | 7501 |
| PavlovianBias | linear | linear | no | no | no | yes | 7542 |
| Combined | log | linear | yes | no | yes | yes | 6949 |

Model comparison was performed by comparing a baseline model to a model in which one ‘component’ was modified. The model we report from includes all the ‘components’ that improved fit compared to the baseline model. The function of the impact of reward and aversiveness onto drift rate was assumed to be linear or logarithmic, and were assessed on whether model fit was improved when including a dummy coded variable that indicated whether the offered value of reward (Dreward) or aversiveness (Daverse) was 0 (D=1) or not (D=0). Conflict was measured as the absolute difference in reward and aversiveness and was estimated to influence the decision threshold parameter. PavlovianBias included information on whether approaching (avoiding) offers involved pushing (pulling) the joystick to respond (PavlovianBias = 1) or vice-versa (PavlovianBias = 0). Lower values of DIC indicate better fit to data. DIC = deviance information criterion.


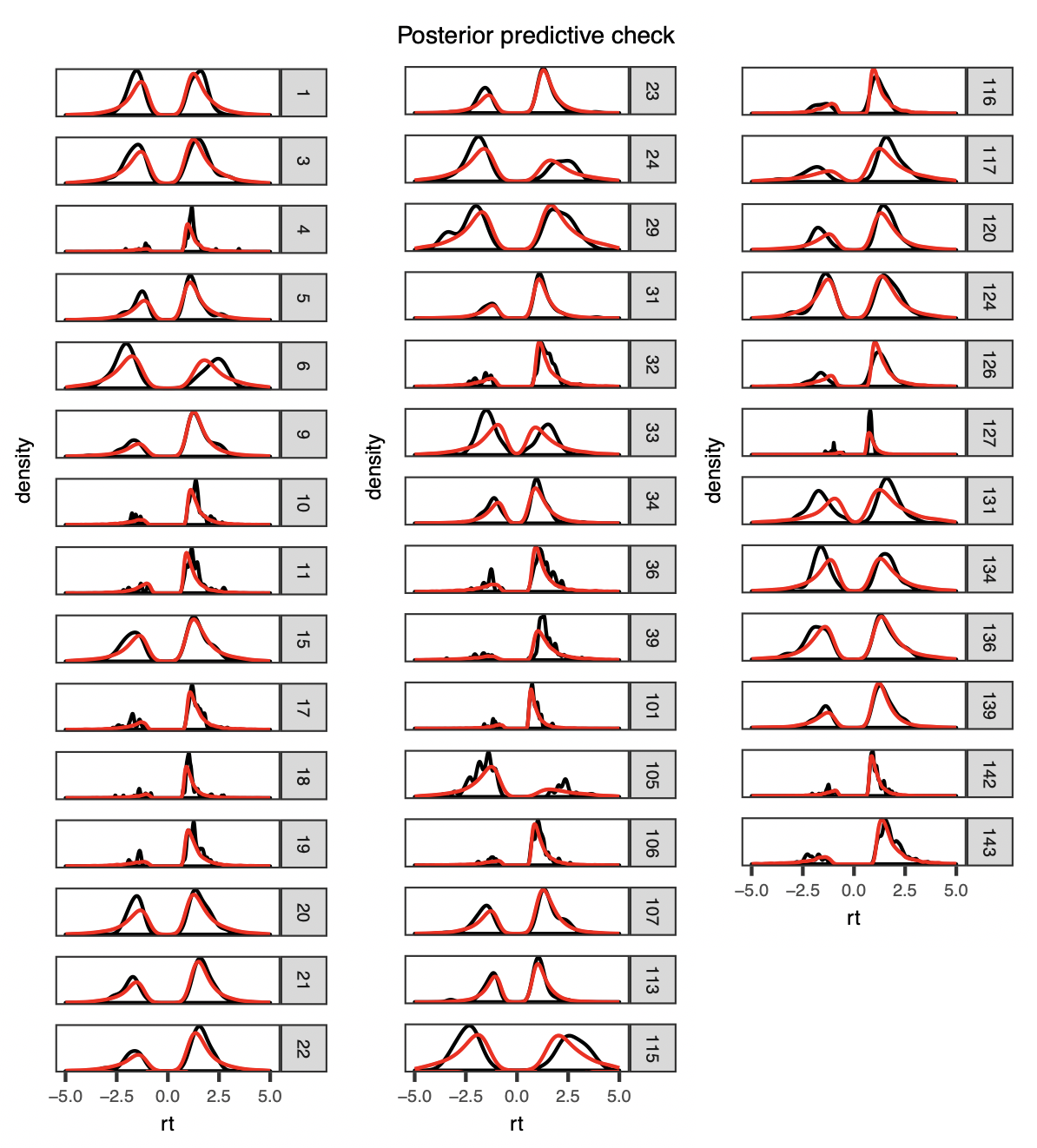


**Fig B.** Observed (black) and predicted (red) response time distributions across subjects. Avoid-decisions are set to be negative to separate RT distributions for decisions to approach and avoid.

**Table B.** Posterior distributions for group parameters at follow-up.


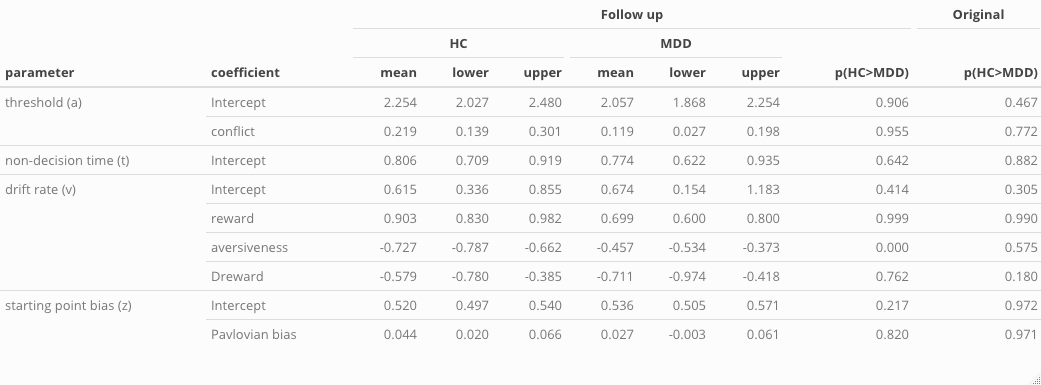


Lower and upper represent the lower and upper bound of the 95% highest density interval of the posterior distribution. For comparison to results from the original dataset, the rightmost column represents probabilities of group difference from original dataset.

## Table C. Multivariate regression for association between clinical measures collected at time of testing and at 6-month follow-up to decision parameters.

|  | **BDI (Beck’s Depression Inventory)** | | | |
| --- | --- | --- | --- | --- |
| *Predictors* | *Estimates* | *CI* | *Statistic* | *p* |
| (Intercept) | 44.08 | 20.97 – 67.19 | 4.20 | **0.001** |
| starting_point | 14.79 | -21.09 – 50.67 | 0.91 | 0.384 |
| drift_rate_reward | -10.56 | -33.16 – 12.04 | -1.03 | 0.326 |
| starting_point_pavlovian_bias | -26.76 | -179.16 – 125.65 | -0.39 | 0.707 |
| starting_point_accumbens | 643.41 | -1104.28 – 2391.10 | 0.81 | 0.435 |
| drift_rate_pACC | -85.98 | -356.87 – 184.90 | -0.70 | 0.499 |
| Observations | 17 | | | |
| R^2^ / R^2^ adjusted | 0.238 / -0.109 | | | |

|  | **MASQ_AA (Mood and Anxiety Symptom Questionnaire – Anxious Arousal)** | | | |
| --- | --- | --- | --- | --- |
| *Predictors* | *Estimates* | *CI* | *Statistic* | *p* |
| (Intercept) | 33.23 | 14.22 – 52.24 | 3.81 | **0.002** |
| starting_point | 4.18 | -27.26 – 35.62 | 0.29 | 0.777 |
| drift_rate_reward | -12.82 | -34.22 – 8.58 | -1.31 | 0.216 |
| starting_point_pavlovian_bias | 33.40 | -111.22 – 178.02 | 0.50 | 0.624 |
| starting_point_accumbens | -283.20 | -1749.36 – 1182.97 | -0.42 | 0.681 |
| drift_rate_pACC | 36.93 | -184.07 – 257.94 | 0.36 | 0.722 |
| Observations | 18 | | | |
| R^2^ / R^2^ adjusted | 0.128 / -0.235 | | | |

|  | **MASQ_GDD (Mood and Anxiety Symptom Questionnaire – General Distress Depression)** | | | |
| --- | --- | --- | --- | --- |
| *Predictors* | *Estimates* | *CI* | *Statistic* | *p* |
| (Intercept) | 53.43 | 31.36 – 75.51 | 5.33 | **<0.001** |
| starting_point | 21.03 | -15.87 – 57.94 | 1.25 | 0.236 |
| drift_rate_reward | -18.78 | -43.92 – 6.36 | -1.64 | 0.128 |
| starting_point_pavlovian_bias | -12.84 | -180.67 – 154.98 | -0.17 | 0.869 |
| starting_point_accumbens | 119.68 | -1664.83 – 1904.20 | 0.15 | 0.885 |
| drift_rate_pACC | 12.01 | -267.30 – 291.32 | 0.09 | 0.926 |
| Observations | 17 | | | |
| R^2^ / R^2^ adjusted | 0.331 / 0.027 | | | |

|  | **MASQ_AD (Mood and Anxiety Symptom Questionnaire – Anhedonic Depression)** | | | |
| --- | --- | --- | --- | --- |
| *Predictors* | *Estimates* | *CI* | *Statistic* | *p* |
| (Intercept) | 96.46 | 72.11 – 120.81 | 8.63 | **<0.001** |
| starting_point | 3.65 | -36.63 – 43.93 | 0.20 | 0.847 |
| drift_rate_reward | -13.00 | -40.41 – 14.41 | -1.03 | 0.322 |
| starting_point_pavlovian_bias | -11.70 | -196.96 – 173.55 | -0.14 | 0.893 |
| starting_point_accumbens | -98.23 | -1976.32 – 1779.86 | -0.11 | 0.911 |
| drift_rate_pACC | 11.37 | -271.72 – 294.47 | 0.09 | 0.932 |
| Observations | 18 | | | |
| R^2^ / R^2^ adjusted | 0.123 / -0.242 | | | |

|  | **HAMD (Hamilton Depression Rating Scale)** | | | |
| --- | --- | --- | --- | --- |
| *Predictors* | *Estimates* | *CI* | *Statistic* | *p* |
| (Intercept) | 13.61 | 3.94 – 23.28 | 3.07 | **0.010** |
| starting_point | -5.12 | -21.12 – 10.88 | -0.70 | 0.499 |
| drift_rate_reward | 0.72 | -10.17 – 11.60 | 0.14 | 0.888 |
| starting_point_pavlovian_bias | -7.05 | -80.63 – 66.53 | -0.21 | 0.838 |
| starting_point_accumbens | -109.87 | -855.84 – 636.10 | -0.32 | 0.754 |
| drift_rate_pACC | 20.64 | -91.80 – 133.09 | 0.40 | 0.696 |
| Observations | 18 | | | |
| R^2^ / R^2^ adjusted | 0.049 / -0.348 | | | |

|  | **PSS (Perceived Stress Score)** | | | |
| --- | --- | --- | --- | --- |
| *Predictors* | *Estimates* | *CI* | *Statistic* | *p* |
| (Intercept) | 44.47 | 32.51 – 56.43 | 8.18 | **<0.001** |
| starting_point | -6.38 | -23.18 – 10.41 | -0.84 | 0.421 |
| drift_rate_reward | -14.74 | -28.57 – -0.90 | -2.34 | **0.039** |
| starting_point_pavlovian_bias | 27.29 | -63.05 – 117.62 | 0.66 | 0.520 |
| starting_point_accumbens | -216.59 | -1011.12 – 577.94 | -0.60 | 0.561 |
| drift_rate_pACC | 55.19 | -62.95 – 173.33 | 1.03 | 0.326 |
| Observations | 17 | | | |
| R^2^ / R^2^ adjusted | 0.454 / 0.206 | | | |

|  | **BDI_followup (Beck’s Depression Inventory at 6-month followup)** | | | |
| --- | --- | --- | --- | --- |
| *Predictors* | *Estimates* | *CI* | *Statistic* | *p* |
| (Intercept) | 18.18 | -8.46 – 44.81 | 1.54 | 0.157 |
| starting_point | -20.93 | -67.43 – 25.57 | -1.02 | 0.335 |
| drift_rate_reward | -14.48 | -45.60 – 16.64 | -1.05 | 0.320 |
| starting_point_pavlovian_bias | -35.31 | -254.62 – 184.00 | -0.36 | 0.724 |
| starting_point_accumbens | -1920.94 | -4297.58 – 455.70 | -1.83 | 0.101 |
| drift_rate_pACC | 109.64 | -217.55 – 436.82 | 0.76 | 0.468 |
| Observations | 15 | | | |
| R^2^ / R^2^ adjusted | 0.347 / -0.015 | | | |

|  | **MASQ_GDD_followup (Mood and Anxiety Symptom Questionnaire – General Distress Depression at 6 month followup)** | | | |
| --- | --- | --- | --- | --- |
| *Predictors* | *Estimates* | *CI* | *Statistic* | *p* |
| (Intercept) | 33.99 | 9.79 – 58.20 | 3.18 | **0.011** |
| starting_point | -19.25 | -61.52 – 23.01 | -1.03 | 0.330 |
| drift_rate_reward | -20.61 | -48.90 – 7.68 | -1.65 | 0.134 |
| starting_point_pavlovian_bias | -98.30 | -297.64 – 101.04 | -1.12 | 0.294 |
| starting_point_accumbens | -2054.66 | -4214.94 – 105.63 | -2.15 | 0.060 |
| drift_rate_pACC | 107.17 | -190.23 – 404.57 | 0.82 | 0.436 |
| Observations | 15 | | | |
| R^2^ / R^2^ adjusted | 0.532 / 0.273 | | | |

|  | **MASQ_AA_followup (Mood and Anxiety Symptom Questionnaire – Anxious Arousal at 6 month followup)** | | | |
| --- | --- | --- | --- | --- |
| *Predictors* | *Estimates* | *CI* | *Statistic* | *p* |
| (Intercept) | 26.82 | 9.80 – 43.83 | 3.56 | **0.006** |
| starting_point | -14.75 | -44.46 – 14.97 | -1.12 | 0.291 |
| drift_rate_reward | -14.81 | -34.69 – 5.08 | -1.68 | 0.126 |
| starting_point_pavlovian_bias | 16.42 | -123.73 – 156.57 | 0.27 | 0.797 |
| starting_point_accumbens | -923.69 | -2442.46 – 595.07 | -1.38 | 0.202 |
| drift_rate_pACC | 132.68 | -76.40 – 341.76 | 1.44 | 0.185 |
| Observations | 15 | | | |
| R^2^ / R^2^ adjusted | 0.402 / 0.070 | | | |

|  | **MASQ_AD_followup (Mood and Anxiety Symptom Questionnaire – Anhedonic Depression at 6 month followup)** | | | |
| --- | --- | --- | --- | --- |
| *Predictors* | *Estimates* | *CI* | *Statistic* | *p* |
| (Intercept) | 67.54 | 35.51 – 99.56 | 4.77 | **0.001** |
| starting_point | -33.20 | -89.12 – 22.71 | -1.34 | 0.212 |
| drift_rate_reward | -0.57 | -37.99 – 36.85 | -0.03 | 0.973 |
| starting_point_pavlovian_bias | -175.92 | -439.64 – 87.79 | -1.51 | 0.166 |
| starting_point_accumbens | -1792.15 | -4650.03 – 1065.73 | -1.42 | 0.190 |
| drift_rate_pACC | 25.03 | -368.40 – 418.46 | 0.14 | 0.889 |
| Observations | 15 | | | |
| R^2^ / R^2^ adjusted | 0.385 / 0.043 | | | |

|  | **HAMD_followup (Hamltion Depression Rating Scale at 6 month followup)** | | | |
| --- | --- | --- | --- | --- |
| *Predictors* | *Estimates* | *CI* | *Statistic* | *p* |
| (Intercept) | 5.03 | -11.69 – 21.74 | 0.68 | 0.513 |
| starting_point | -21.10 | -50.28 – 8.08 | -1.64 | 0.136 |
| drift_rate_reward | -3.57 | -23.10 – 15.95 | -0.41 | 0.689 |
| starting_point_pavlovian_bias | -7.25 | -144.86 – 130.35 | -0.12 | 0.908 |
| starting_point_accumbens | -1495.25 | -2986.47 – -4.04 | -2.27 | **0.050** |
| drift_rate_pACC | 83.26 | -122.03 – 288.55 | 0.92 | 0.383 |
| Observations | 15 | | | |
| R^2^ / R^2^ adjusted | 0.397 / 0.062 | | | |

|  | **PSS_followup (Perceived Stress Score at 6 month followup)** | | | | |
| --- | --- | --- | --- | --- | --- |
| *Predictors* | *Estimates* | *CI* | | *Statistic* | *p* |
| (Intercept) | 26.75 | 9.41 – 44.09 | | 3.56 | **0.007** |
| starting_point | -17.99 | -48.32 – 12.33 | | -1.37 | 0.208 |
| drift_rate_reward | -12.77 | -33.47 – 7.92 | | -1.42 | 0.192 |
| starting_point_pavlovian_bias | -35.03 | -177.27 – 107.22 | | -0.57 | 0.586 |
| starting_point_accumbens | -1607.26 | -3222.46 – 7.93 | | -2.29 | 0.051 |
| drift_rate_pACC | 186.00 | -48.49 – 420.49 | | 1.83 | 0.105 |
| Observations | 14 | | | | |
| R^2^ / R^2^ adjusted | 0.498 / 0.184 | | | | |
|  | **SHAPS_DIM** | | | | |
| *Predictors* | *Estimates* | *CI* | *Statistic* | *p* |  |
| (Intercept) | 29.87 | 21.88 – 37.86 | 8.14 | **<0.001** |  |
| starting_point | -8.60 | -21.82 – 4.62 | -1.42 | 0.182 |  |
| drift_rate_reward | 2.27 | -6.72 – 11.26 | 0.55 | 0.592 |  |
| starting_point_pavlovian_bias | -13.80 | -74.58 – 46.99 | -0.49 | 0.630 |  |
| starting_point_accumbens | 29.63 | -586.63 – 645.89 | 0.10 | 0.918 |  |
| drift_rate_pACC | 25.65 | -67.24 – 118.55 | 0.60 | 0.559 |  |
| Observations | 18 | | | |  |
| R^2^ / R^2^ adjusted | 0.230 / -0.091 | | | |  |

|  | **SHAPS_CAT** | | | |
| --- | --- | --- | --- | --- |
| *Predictors* | *Estimates* | *CI* | *Statistic* | *p* |
| (Intercept) | 3.58 | -3.24 – 10.40 | 1.14 | 0.275 |
| starting_point | -7.27 | -18.56 – 4.01 | -1.40 | 0.186 |
| drift_rate_reward | 1.95 | -5.73 – 9.63 | 0.55 | 0.590 |
| starting_point_pavlovian_bias | 1.58 | -50.33 – 53.48 | 0.07 | 0.948 |
| starting_point_accumbens | 64.85 | -461.36 – 591.05 | 0.27 | 0.793 |
| drift_rate_pACC | 3.68 | -75.64 – 82.99 | 0.10 | 0.921 |
| Observations | 18 | | | |
| R^2^ / R^2^ adjusted | 0.226 / -0.097 | | | |

## Table D. Intraclass correlation coefficient for individual parameters across sessions.

##
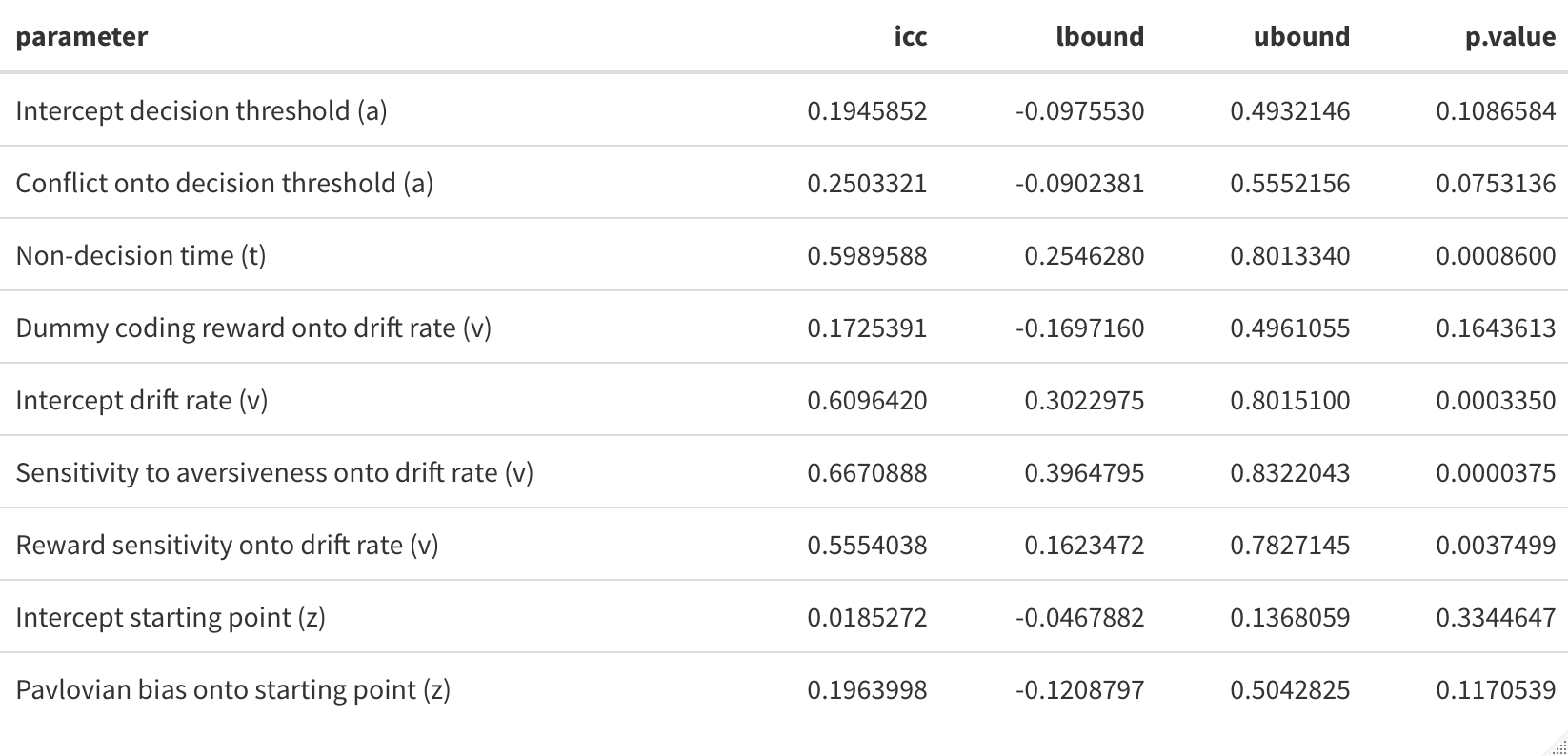


## References

1. First MB, Spitzer RL, Gibbon M, Williams JBW (2002): Structured clinical interview for DSM-IV-TR axis I disorders, research version, patient edition. SCID-I/P.

2. Hamilton M (1960): A rating scale for depression. J Neurol Neurosurg Psychiatry. 23: 56.

3. Beck AT, Steer RA, Brown GK (1996): Beck Depression Inventory-II (BDI-II). Psychol Corp.

4. Snaith RP, Hamilton M, Morley S, Humayan A, Hargreaves D, Trigwell P (1995): A scale for the assessment of hedonic tone the Snaith-Hamilton Pleasure Scale. Br J Psychiatry. 167: 99–103.

5. Ottenbreit ND, Dobson KS (2004): Avoidance and depression: the construction of the cognitive-behavioral avoidance scale. Behav Res Ther. 42: 293–313.

6. Watson D, Weber K, Assenheimer JS, Clark LA, Strauss ME, McCormick RA (1995): Testing a tripartite model: I. Evaluating the convergent and discriminant validity of anxiety and depression symptom scales. J Abnorm Psychol. 104: 3–14.

7. Cohen S, Kamarck T, Mermelstein R (1994): Perceived stress scale - Measuring stress: A guide for health and social scientists. Oxford University Press New York, NY, 235–283.
