# Supplementary material for: Computational phenotyping of brain-behavior dynamics underlying approach-avoidance conflict in major depressive disorder
Source: PLoS Comput Biol. 2021 May 10;17(5):e1008955. doi: 10.1371/journal.pcbi.1008955 (PMC8136861; doi:10.1371/journal.pcbi.1008955)
Supplement: Attachment — Submitted filename: Comments to the Authors.docx [file pcbi.1008955.s002.docx]

**Comments to the Authors:
Please note here if the review is uploaded as an attachment.**

Reviewer #1: This interesting manuscript uses a drift-diffusion model where both aspects of stimulus value and BOLD activation in two brain regions, the caudate nucleus and pACC, are used to determine drift rates. In addition, the decision threshold multiplicatively depends on subthalamic activity and the absolute difference between rewarding and appetivive value aspects of stimuli, while Pavlovian bias multiplies nucleus accumbens activity to determine Pavlovian bias, here mapped to the starting point of the diffusion process. The model is used to characterise two samples, of 18 depressed, unmedicated (MDD) and 25 health control (HC) participants. The authors find that using both computational parameters and brain measures, but most importantly the former, they can classify the 1/3 held-out sample they use with an AOC of 68%. They also find that MDD participants differ from HC with respect to some computational parameters esp. the weight of reward (but not aversiveness) on the drift rate.

I hesitated to recommend major revision rather than rejection of this study because the very small sample of 18 MDD participants really does not allow conclusions to be drawn about MDD itself. However I felt that the methodology is intersting and the results should be published, so that they can be aggregated with similar studies and conclusions be drawn about this interesting group of disorders. This is especially important as MDD is likely to be a heterogenous group of disorders, and therefore small subsamples from this population may differ substantially from each other. The authors do recognise that the sample is small, but in my view this is a very important limitation. Really, the sample size is so small that holding out 1/3 means that predictive testing was only done on 6 depressed participants (and a proportionate number of controls). Therefore the 68% AOC should be regarded as a proof-of-principle analysis. Really, there is no grounds at all to suggest that the results point to clinical utility.

We agree with the reviewer that the small sample limits the degree to which we can extend beyond this sample, and thus whether the results point to clinical utility. In addition to bolstering the results with new independent analysis (please see below), we have made changes throughout the manuscript to reflect that these results show the potential utility of our approach, but that additional studies are needed to show clinical utility.

I am aware of how difficult it is to recruit unmedicated patients with major depressive disorder that don't meet exclusion criteria of all sorts in a practical length of time, for a single laboratory. This is why I do not recommend rejection of the paper. However, as the most predictive aspects of the modelling are the computational parameters, and hence recruiting a second lot of 25+25 participants for behaviour-only analysis with this one task may be feasible in a relatively short time, maybe tagging the task on MDD-HC studies.

While we are unable to collect a new dataset at this time (especially due to COVID-19), we have addressed this issue by testing the degree to which our model parameter estimates and classification results generalize beyond the initial data we had analyzed in the original manuscript. Specifically, 6 months after the original study, 10 patients with MDD and 17 healthy controls participated in a follow-up behavioral session. We have now applied the same computational model (without neural regressors) to this follow-up session. Importantly, there was a significant intraclass correlation between individual parameter estimates in the two sessions for all parameters except one, indicating that the modeling parameters were reliable. This analysis further confirmed the effect of reduced reward sensitivity onto drift rate in MDD in the follow-up data, providing support for the generalizability of this effect. Some of the other findings from the original dataset were not as strong. We discuss reasons for this discrepancy, which could be attributed to the effect of having performed the task before, the lower sample size for the follow-up assessment, or other reasons. Finally, we show that the classifier trained and tested on the original dataset has value. Specifically, in a completely out of sample test, we found that the classifier on the original dataset can predict disease status based on the parameters fit from the follow-up data with a 62% accuracy (AUC = 0.55). Considering that the neural regressors were not included in the modeling of the follow-up data, we deem these classification results promising. Further, as described in the manuscript, three of the MDD participants were remitters at 6-month follow up, with symptom scores on the Hamilton Depression Rating Scale below 7, and an additional participant with a score of 8. Due to the low sample size, these were included in the analysis. However, although optimistically and more speculative, categorizing these three (four) as controls improved classification accuracy to 69 (73) % (AUC=59 (61)). These new analyses and findings are reported on pp. 28-31 of the revised manuscript

The authors mention computational model comparison way too briefly in the manuscript, and even refer to another paper as details of the sample. As a reviewer, I found this very frustrating. Essentially, if the paper was to be published like this it would say to readers 'trust our reviewers, they went into the trouble to find out if the winning model really is better, if the excluded participants are likely to bias the results, etc.'. I think there should be clear evidence about model comparison in the results, good information about the sample and exclusions in the supplement, and discussion of both of these in the Discussion.

We agree that these points were not sufficiently explained or discussed. In the revised manuscript, we have therefore included more information about the sample and exclusion criteria in the methods section (page 9), more information about the sample in the supplement (page 2), and more information about model comparison in both the methods (pages 14-18) and results (pages 20-21). Lastly, the low sample size and potential for alternative better models are now more thoroughly discussed in the discussion.

Reviewer #2: -This is a well-motivated and well-written paper, reporting results of sophisticated computational and Bayesian analyses of potential clinical relevance. While I am enthusiastic about the study, the paper has some limitations that should be addressed before publication – mainly having to do with clarity of methods/results. I expand on these issues below (many of which are minor), mainly in order of presentation in the text. I should note that several appear to relate to the fact that the methods were moved to the end, and the authors failed to define/explain things the first time they appear in the manuscript as it is currently organized.

We thank the reviewer for the thorough and comprehensive comments. We agree that many of the issues reported here result from having the methods section after the discussion. We have therefore moved the methods section (back) up so that it now directly follows the introduction. We believe this substantially improved clarity.

-Around line 97 – The authors should here define what “reward sensitivity” means (as they use it in the paper) before using it below in a way that assumes the reader knows what it refers to.

We believe the reviewer is referring to the use of reward sensitivity in the abstract (but we seem to have different line numbers so cannot be sure). We have now defined reward sensitivity in the abstract. Specifically, we now state:

«MDD was associated with reduced reward sensitivity, measured as decreased impact of reward onto evidence accumulation»

-The acronym RT should be defined on first use.

Fixed.

-The authors mention pavlovian-instrumental transfer without explaining what it refers to, which is necessary for understanding how it connects to their own work as the authors mention.

This is now explained in the Introduction (page 6):

«MDD has also been linked to alterations in Pavlovian-Instrumental-Transfer, which captures the influence of background Pavlovian valence on instrumental decision making»

-There are some potential (minor) issues with consistent terminology that may cause the reader some unnecessary confusion. For example, it appears the authors use “evidence accumulation” and “drift rate” as synonymous in certain places, where sticking with consistent terminology would be clearer (e.g., perhaps initially discuss how drift rate controls the speed of evidence accumulation, but then always refer to the actual drift rate parameter after that point).

Thanks for noticing this. We have gone through the manuscript to make the relationship between drift rate and evidence accumulation more clear, specifically referring to evidence accumulation before the parameters are introduced, and when introducing drift rate specifying that it captures the speed of evidence accumulation.

-The authors state that their measures were “sufficient to classify individual patient status”. Without qualification (and/or stating how well classification performed above chance), this read overly strong to me.

We agree that the phrasing implied better performance than we reported; to address this important point, we have changed this sentence to: «computational biomarkers aided in classifying individual patient status». We have also now bolstered the classification results by applying them to the follow-up dataset without further training (please see response to R1 above).

-Figure 1 – The parameter variables should be added to the legend. At the moment, the reader can’t easily map the variables in the figure (e.g., z) to their names in the legend. Also, all parameters are illustrated except for non-decision time. It’d be nice if that could be included in the DDM illustration for completeness.

Thanks for the excellent suggestion. As recommended, the figure now includes an illustration of non-decision time and the parameter variables are included in the legend.

-Around line 160 - The authors assume a lot of reader knowledge about modeling methods. They should say something more about their model estimation and comparison methods (e.g., MCMC, what chains are, etc.). Some of this also isn't thoroughly explained in the methods at the end either.

We are grateful to the reviewer for highlighting areas that required additional clarifications. We have revised these sections to increase clarity, including by adding more details regarding model estimation and model comparison methods in both the Methods and Result sections (pages 14-18 and 20-21).

-They provide little task detail, and not much more in the methods. More details are needed, and the reader shouldn’t need to go digging in previous manuscripts to understand basic aspects of the task (e.g., how many trials? What was stimulus aversiveness based on? How many different stimulus aversiveness and reward value combinations were used, and how many times each? How exactly was push-pull and approach-avoid orthogonalized in terms of trial order? etc.).

The reviewer is raising several legitimate points, and we apologize for the original omissions. We have now updated the description of the task to include the number of trials (105), how aversive stimuli were classified (normed ratings from Lang et al., 2008), the number of values of aversive (6) and rewarding (16) stimuli and how the response mapping was orthogonalized (random and counterbalanced).

-Figure 3 – The legend refers to panels A, B, C, etc. However, these labels are nowhere in the figure. Also, the Y axes are unclear, both in terms of units and labels. Using the parameter names (e.g., “drift rate”) would be clearer, and/or they should state what the variables (e.g., z) refer to in the legend. In general, I felt like many figure details were

not explained in the legend.

We have added letters to the panels, and the legend now explains what the variables refer to.

-Line 239 – It seems like a citation for this statement is missing.

Thanks for catching this. The citations have now been included.

-Discussion of the STN seems to come out of nowhere in the results, and the acronym is never defined. Discussion of its relevance should be in the introduction.

The introduction did include reference to the STN, but without the abbreviation. This is now fixed.

-The authors repeatedly mention a plausible role for the midcingulate. It made me wonder why this wasn’t included as an ROI.

Previous studies have found a role for the midcingulate in signaling to the subthalamic nucleus (STN) the need to extend decision boundaries. Therefore, we chose not to include the midcingulate as a neural measure in an attempt to limit the number of neural ROIs, which could lead to false positives, and given that the effect seen in the midcingulate should be reflected in STN.

-The acronym AUC is never defined and used only once in the text.

Thank you for the careful review. The abbreviation is now included at first use.

-The authors report AUC for the model with poor classification (showing it performs at chance levels), but do not show the AUC supporting above chance performance for the biomarker model. Both should be reported for consistency.

As recommended, the revised text now includes AUC values for both classifiers.

-Figure 4 and related text – The authors could help the unfamiliar reader understand some of this better. For example, do their reported AUC values indicate high classification accuracy? Or above chance but still fairly low? Some more intuitive indication of “how much” above chance their model did would be helpful. Similarly, the authors do not help the reader to understand how to interpret the beta values in this context. I think many readers will walk away from this figure (and associated text) fairly unclear about exactly how good classification was. They also need to define figure acronyms in the legend.

We appreciate that the AUC was not explained well enough. We have included an explanation of how to interpret the AUC, saying ‘the AUC statistic can be interpreted to measure the probability of correctly classifying two randomly drawn samples from each the two classes, or alternatively, it is the true positive rate averaged across all possible values of false positives’, in both the methods (p.19) and the results (p. 26). We have also updated the legend for figure 4 to explain acronyms and to better explain what the beta weights represent:

«Figure 4. Classification of MDD status and feature importance for the computational biomarker classifier. A, the receiver operating characteristic (ROC) curve and the ROC area under curve (AUC) statistic for a classifier using individual parameter values from the computational model (purple) and a classifier using mean observed behavioral measures of response time and approach rate and mean activity in ROIs (caudate nucleus, nucleus accumbens (NAcc), pregenual anterior cingulate cortex (pACC), and subthalamic nucleus (STN)). B, mean estimated beta-coefficients from classifier with 95% confidence intervals for the classifier using computational biomarkers (purple). Coefficients are sorted by weight from left to right as the absolute distance from 0, the magnitude of which indicates the importance of each feature for the classification. Int = Intercept, PavBias = Pavlovian bias, v = drift rate, z = starting-point bias.»

-The findings with clinical measures were often only borderline significant. It’d be nice to have a sense of the effect size here. Also, why not stick with the Bayesian approach here? Can you show something like a Bayes factor score that provides clear evidence for these effects?

We have updated these statistics to also report the confidence interval on the beta-coefficients to help give a sense of the size of the effect:

«This analysis demonstrated that reward sensitivity (*v-*reward) was negatively associated with perceived stress (*b*= -14.74 (CI = -28.57 – -0.90), *t*(7) = -3.04,  *p* = .039), and the individual impact of the nucleus accumbens on starting point was associated with 6-month follow-up scores on the Hamilton Depression Rating Scale (*b*= -1495 (CI = -2986.47 – -4.04), *t*(8) = -2.4, *p* = .04). These findings further reify the utility of the computational biomarkers for predicting symptom progression.»

-Around line 341 – They mention their analyses could be extended to anxious populations. It made me wonder why only to anxiety? Is this not potentially relevant for other psychiatric disorders as well?

As correctly pointed out by the reviewer, abnormal approach-avoidance decision making has been implicated in several neuropsychiatric disorders, including mood and anxiety disorders, eating disorders, substance use disorders, and personality disorders (see Loijen et al., 2020 for a recent review) This additional information (and references) have been added to the Discussion (see page 32)

-Around line 349 – The authors mention direct links to anhedonia. It made me wonder about possible ways to test whether their measures correlate with standard experimental measures of anhedonia (e.g., SHAPS). If the authors expected this connection with their model, why not include such measures?

Thank you for noticing this oversight. We do have clinical measures of anhedonia, specifically the Snaith-Hamilton Pleasure Scale (SHAPS). We now include these measures in the regressions, but do not find a significant association to any of the model parameters. This lack of finding is discussed as a caveat in the discussion where reward sensitivity is linked to anhedonia:

«Reduction in reward sensitivity in MDD has been found in other tasks, including instrumental learning [27], and directly maps onto anhedonia, an important endophenotype of depression [55]. However, in the current study we did not find our measure of reward sensitivity to be related to Snaith Hamilton Pleasure Scale [56], a self-report measure of anhedonia.»

-Line 363 – there is a missing word here.

Thank you for catching this, we have now added ‘with MDD’ to the following sentence:

«Finally, response mapping had opposite effects in individuals with MDD and controls, whereby pushing a joystick to approach (slightly) increased starting point to avoiding offers for MDD and approaching offers in healthy controls.»

-Throughout the discussion, I felt like there was a lot of re-stating results and fairly little actual interpretation. Do the authors not have some proposal about what their results mean (either mechanistically or clinically)? For example, how should we think about nucleus accumbens having opposite influences in each group? Or what might it mean that there is a reversed pavlovian effect?

We agree that there was a general lack of discussion of the effects. To address this important point, we have extended these sections to include potential explanations and interpretations of the reported effects in the Discussion section.

-The limitations section discusses things that aren't limitations. It mainly talks about results they expected to find but didn’t, which says nothing about limitations in their methods directly. They also don’t mention limitations they could. For example, the sample size is quite small. Do power limitations prevent interpretation of false negatives? Also, the sample was female – which prevents generalizing these results to any depressed men.

Again, we agree that the small sample size did not receive enough attention in the limitation, and that some of the other limitations instead were reflections on why we did not find hypothesized effects. These latter descriptions have now been moved up to the general discussion, while the limitations now include more focus on the small sample size (while also highlighting the addition of the follow-up dataset) and also describes that generalizing these effects to men is difficult.

-It felt like a conclusions section was missing after the limitations.

As recommended, we have now included a short conclusion section at the end of the discussion (page 34), which reads:

«Computational modeling revealed that participants with MDD solved approach-avoidance conflict differently than healthy controls. In particular, MDD was associated with reduced reward sensitivity. Individual parameters were linked to clinical measures of MDD and were useful for classifying diagnosis. Collectively, these findings establish the promise of computational psychiatry approaches to dissecting approach-avoidance decision dynamics relevant for affective disorders.»

-Their methods refer the reader to other papers a lot, where understanding the study requires many of these details (especially in relation to the task, as mentioned above).

The reviewer is raising a legitimate point. In the revised manuscript, we have extended the Methods in an attempt to avoid forcing the reader to check other papers to understand methodological details.

-I felt like the authors could expand on how to think about the influence of neural activity on parameters. It’s a bit complex, because these reflect different levels of description – where one assumes that computational mechanisms/parameters are implemented by (i.e., correspond to) something within neural hardware. But here it’s not that a given ROI is the thing that implements a decision parameter, but that it influences it in some way. So should this be thought of as that ROI affecting a different brain region/process? Or how should this be thought about?

This is a good point. Normally computational models are fit to behavior alone which assumes that the processes are stationary across all trials – and here we are asking whether the neural measures are likely to implement the parameter because if variation in the signaling correlates with the parameter then it changes decision making. However, we do not assume that the ROI solely implements the parameter but rather there is a network that does so, and we are asking whether and how the ROI contributes. We have added a section in the methods to explain these assumptions. Specifically, on page. 15, we now state:

«After establishing the best-fitting model to average behavior, we augmented the model to determine whether decision parameters are altered on a trial-by-trial basis as a function of BOLD activity in the pACC, caudate nucleus, nucleus accumbens and subthalamic nucleus, and to assess how this impact differed across groups. Estimating the impact of neural correlates on decision parameters quantifies how ROIs are linked to mechanistically meaningful parameters, over and above the effect of behavioral manipulation.»

-Around line 449 – They state they used priors informed by previous work. But what were they?

The HDDM package includes default priors, which are informed by a wide range of empirical studies but are sufficiently conservative to allow for deviations in mean parameters based on the data. We have added more detail to explain this in the text (page 16-17):

«For baseline/intercept-parameters we used priors from the HDDM package, which are informed by a wide range of empirical studies but are sufficiently conservative to allow for deviations in mean parameters based on the data [41]. Slope-coefficients used noninformative priors centered at 0. Intercept-parameters were estimated separately for each participant, while other coefficients were estimated on a group level, due to the inherent noise in neural signals. All predictor variables, with the exception of dummy-coded variables, were z-transformed prior to analysis. »

-I was a bit unclear about their description of model comparison. It says they added one component and then compared to baseline for each possible component. But components can interact. So did they not compare models with different combinations of included components?

Thank you for highlighting the need to clarify our approach. To address this important point, we have revised the description of the methods we used for model comparison. The reviewer is correct in that we identified components that increased model fit by comparing to a baseline model. We did not consider all possible factorial interactions between model parameters to avoid an explosion in the model space, and because we are taking a relatively theory-driven approach and attempted to avoid selecting overly complex models. Thus, we took a more systematic approach to determine whether adding parameters on top of baseline models can improve fit (see e.g. Guitart-Masip et al 2012; Cavanagh et al 2013; Swart et al 2017; 2018, among others for a similar approach).

-In summary, all of these points should be addressable and most reflect issues of clarity/completeness. I hope the authors find them helpful in improving the manuscript.

The insightful comments we received were indeed instrumental for providing additional clarification and elaborations. We are very grateful to the reviewer for the many constructive suggestions.
