# Supplementary material for: Computational phenotyping of brain-behavior dynamics underlying approach-avoidance conflict in major depressive disorder
Source: PLoS Comput Biol. 2021 May 10;17(5):e1008955. doi: 10.1371/journal.pcbi.1008955 (PMC8136861; doi:10.1371/journal.pcbi.1008955)
Supplement: Attachment — Submitted filename: response_letter_2nd_revision.docx [file pcbi.1008955.s003.docx]

Reviewer #1: I thank the authors for their attention to my concerns, and those of the other reviewer, which were also very interesting. Although my original concrerns are appeased, a couple of new questions are now troubling me.

The authors write, l. 307 'To capture potential differences between individuals with MDD and healthy controls we ran the model separately for the two groups, using the same prior distributions.' If this is a hierarchical fit, running the model 'separately for the two groups' must mean that the model infers the group distributions for each group separately, based on the same priors on parameters describing these two distributions. Crucially, the consistency between each empirical prior and the parameters of each participant is also maximized, so that the parameters of each participant are informed indirectly by all the data of the group they belong to, but not the data of the other group. The authors then say (l. 321) that they 'test effects as the posterior distribution of difference between group posterior distributions and report the probability of one group having a higher estimated parameter value as the proportion of the distribution of difference above 0'. From this description, I am concerned whether shrinkage in the separate distributions of the two groups biases the second step towards false positives, as we and others some have shown in simulation studies. I would have thought that the correct way to avoid such an effect of shrinkage would be to include in the same model fitting separate distributions for the two groups, but also fit the difference between the means of these two distributions directly. Then, the credible interval of the difference directly gives what the authors seem to have estimated as a separate step. This is similar to a random effects fit where the data of the one group is allowed to inform the other group but only to the degree that the two groups are the same.

It is true that each group was estimated separately. And while we agree that this enables shrinkage to occur separately in principle that should yield a more accurate estimate of the group distribution, given the assumption that participant parameters are in fact sampled from that group distribution. However we agree that there is a potential for false positives. To test whether this impacted the group differences we re-ran the model with a mixed effect Bayesian hierarchical regression model using the BRMS toolbox built on top of STAN in R (it is not straightforward to do the mixed effect model in HDDM). In that model we directly estimated the group differences in the following form (excluding parameter-specific predictors), as suggested by the reviewer:

1 + group + (1|ID)

The model was in all other respects identical to the two models we had reported in the previous version of the manuscript. The results from this model were strikingly similar to our previous model (see figure below). We therefore chose to keep the results from the original models in the main manuscript, but we report in the Methods that this approach led to similar results, which we include in the supplement.

“To capture potential differences between individuals with MDD and healthy controls we ran the model separately for the two groups, using the same prior distributions. We also modeled data from the two groups together in a mixed-effect regression model to directly estimate their group differences in BRMS, which gave nearly identical results (see Supplementary Figure S1 for results from mixed-effect model).”

**Mixed-effect model**

**Original model analyzing groups separately**

**Top: results from mixed-effect model directly estimating the effect of MDD.**

**Bottom: results from two-models approach. Both estimates, their uncertainty and the effect of group are almost identical with the two approaches.**

Secondly, I am concerned about the longitudinal analysis, and especially the claim that the follow-up data were 'completely out of sample'. I think the expression 'out of sample' is used in a rather different way, that is, that the test data are independent to with the training data (conditioned on group etc. of course). Here, the data are highly correlated by virtue of these being the same individuals. I'm not sure if the authors have done this already, but I think that two forms of out-of-sample prediction have to be distinguished here. The first is test-retest reliability, which is what the authors are doing. The same classifier that classifiers a participant at baseline also classifies them the same way at follow up. The second is true out-of-sample classification, where each follow-up datum is tested with a classifier *excluding that individual at all points where the classifier is trained*.

We agree with the reviewer that our use of the term “out of sample” could be somewhat misleading. Although it was true that the classifier never saw the follow-up data, the reviewer is right that any strong correlations between baseline and follow-up parameters might undermine this claim. We therefore took the suggestion: we trained and tested classification models iteratively in which the individual tested (at follow up) was not included in the training set (baseline data). The performance of the classifier at follow up was somewhat weaker, with prediction accuracy at 59 % accuracy (compared with 62% originally) for the classification using diagnosis at baseline. Nevertheless we continued to find that classification was somewhat improved at test when the three (+1 borderline) remitters were considered to be healthy at follow up (64% and 69% accurate depending on that borderline case). Note that this result in which the classifier improves when classifying remitters as HC (previously and also here), further suggests that the classifier is not just overfitting to individuals. We have updated the manuscript including these new results:

### “Classification

To evaluate the generalization of the classifier distinguishing MDD from HC based on model parameters alone, we tested how well this same classifier (but without the neural regressors) could distinguish MDD from HC based on model parameters at follow-up. Because we had found at baseline that the neural regressors (biomarkers) were helpful for classifying patient status, we expected only moderate success in classifying the follow-up unseen data without such regressors. Nevertheless, we found that the classifier at follow-up predicted diagnosis with an accuracy of 62% (AUC = 0.55). Moreover, considering that three MDD subjects were considered in remission (and one was borderline), this classifier success improved to 69/73% (AUC=0.59/0.61) if these subjects are considered to be healthy (HC) at follow-up. This latter result suggests that the classifier was not simply overfitting to individuals given that the same participants had different clinical status. Nevertheless, to further test whether the performance of this classifier was driven by including the same participant in the trained and tested sample, we also conducted as a purely out-of-sample test in which we iteratively predicted an individual at follow-up that was excluded during the training of the classifier. This approach led to reduced performance (59 % accuracy), but was still found to improve when the three (and one borderline) remitters were considered to be healthy controls at follow-up, with an accuracy of 64% (69%).”

Thirdly, I am puzzled about the results that did not replicate at follow-up, or conversely, the new results that emerged then. Of course the main problem may be the small size of the sample, but I wonder if the authors could also discuss, in the discussion, the issues of state vs. trait and the role of the dimensional character of psychiatric disorder, and of course what happened in the follow-up period of six months. First, I observe that in this period three of the 10 participants who were originally classified as MDD no longer fulfilled such criteria, but they were included in analyses due to the small sample size. At first reading, this means that the classifier picked up some type of trait-like vulnerability or partial remission, as well as criteria-fulfilling MDD, or the results would be stronger without the remitters. Second, although the numbers are so small, one would expect more than 30% remission rate at 6 months - although of course these things vary enormously - but this 30% reminds the reader of the issue of self-selection. The retention rate for MDD is less than 1/2, which is is well below conventional satisfactory follow-up rates. Third, the remissions and composition of the follow-up sample suggest rather strongly that all results should be analysed dimensionally rather than categorically, using a measure of mood suitable for both clinical and health populations (if the authors have one). It would, for example, be very interesting if the classifier predicts status corrected for mood or indeed vice versa. Next, did the patients receive no treatment in these six months? Could it be simply treatment, rather than change of clinical status or randomness, that explains baseline-follow-up results?

The reviewer raises several important and interesting points here.

First, the reviewer is correct that both the retention and remission rates are somewhat low. It could be that these are related and that a process of self-selection is at play, where remitted subjects are less inclined to come in for a second testing. We have updated the Limitations section of the Discussion raising this as a potential concern, including that we did not have control over, or information on, whether participants sought treatment in the interim period between baseline testing and follow-up.

Second, the question of whether performance on the approach-avoidance conflict task is capturing effects of state or trait is an interesting one. Due to the low sample size we cannot confidently draw conclusions in either direction, but we note in the Discussion that the results of improved performance when remitters are categorized as healthy controls hints that it captures state.

Lastly, the suggestion that these effects could be captured dimensionally rather than categorically is an interesting point. We believe that this might indeed be the case, but we do not have a good metric of mood in which healthy controls show sufficient variability, and a linear classification likely would require a larger sample size.

All of these issues have now been included in the Limitations section of the Discussion:

“We found that individually estimated model parameters were related to clinical measures in individuals with MDD and predicted future status. However, larger samples are needed to confirm these promising results of identifying computational biomarkers of approach-avoidance conflict decision making in MDD. In addition, despite showing that the classifier with computational model parameters outperformed the classifier using behavioral variables and neural correlates, future studies should increase sample size to more reliably estimate the utility of using model parameters to classify MDD status. A larger sample size could also allow testing linear classifiers, using dimensional measures of mood, in contrast to the categorical outcome of healthy vs. MDD used here. Such an approach could describe to which degree whether the group effects observed here reflect effects of state or trait. The current results of improved classification when categorizing remitters as healthy controls hints that it is capturing state. An increased sample size would also more conclusively reveal whether clinical measures of mood not found to be significantly associated with decision parameters in the current study indeed aren’t associated, or whether these results reflect low power to detect such effects. Further, the dataset used here consisted of female participants, thus precluding us from evaluating the generazibility of the findings. We also note that the retention rate and, within MDD, rate of remitters, were somewhat low. This could reflect an issue of self-selection, where remitted participants were more likely to not come in for a second testing. Finally, alternative models and neural circuits beyond those we tested could be shown to provide a better fit to data. Given the sample size we focused only on theoretically motivated ROIs in the computational model.”

Finally, I note that the authors very nicely included SHAPS following the suggestion of the other reviewer, but found no significant results. The authors should report a power analysis which will hopefully reassure us that the null result is simply due to lack of power. Though weak, the result is compatible with a view that sees anhedonia as a symptom of depression without as much interest with respect to vulnerability or endophenotype than the field hoped a few years ago.

In describing these results we now note that the lack of findings could be explained by a low samples size, given that for a moderate effect size more participants would be required for a satisfactory power, see point directly above.

Lastly, we discovered that we had not accurately calculated intraclass correlation coefficients for parameter estimates at the two test points, and have therefore updated the description in the results section (see below) as well as the statistics from these analyses in the supplementary methods.

### “Test-retest reliability of model parameters and replication of reward sensitivity effect

We first assessed the reliability of individual parameter estimates by calculating two-way intraclass correlation coefficients of mean parameter estimates across the two tests. The intercept drift rate, sensitivities to reward and aversiveness onto drift rate and non-decision time parameters were significantly correlated across the two data collection phases (p < 0.05), while the remaining parameters were not (p > 0.05) (see Supplementary Methods for statistics for each parameter). We found that the parameter estimates for starting point bias and decision threshold depended on the inclusion of neural predictors, thus providing a potential answer as to why these parameters did not correlate between time-points.”
